# The first complete mitochondrial genome of the migratory dragonfly *Pantala flavescens* Fabricius, 1798 (Libellulidae: Odonata)

**DOI:** 10.1080/23802359.2021.1882914

**Published:** 2021-03-15

**Authors:** Felix Joke David, Rebecca Herzog, Arne Bielke, Nicole Bergjürgen, Hans-Jürgen Osigus, Heike Hadrys

**Affiliations:** Institute of Animal Ecology, University of Veterinary Medicine Hannover, Foundation, Hannover, Germany

**Keywords:** Mitochondrial genome, Odonata, *Pantala flavescens*, A + T rich control region, migratory insect

## Abstract

*Pantala flavescens* is the world’s most abundant and widely distributed dragonfly and with its outstanding migratory capacity an important model system to study insect migration at the evolutionary base of winged insects. We here report on the first complete mitochondrial genome (mitogenome) of *P. flavescens* sampled from a population in Rufiji River, Tanzania. The mitogenome is 14,853 bp long with an AT-biased base composition (72.7% A + T) and encodes a typical set of 13 protein-coding genes (PCGs), 22 tRNAs, and two rRNAs. The control region (CR) (171 bp) is the shortest reported in any anisopteran odonate, so far. Phylogenetic analyses support the placement of *P. flavescens* within the Libellulidae.

The migratory odonate *Pantala flavescens* is described as the most successful odonate species in terms of abundance and geographic distribution (e.g. Dijkstra and Clausnitzer 2014). This status is likely achieved by its remarkable migratory capacity with long, multigenerational migration routes of up to 18,000 km. This is the longest known distance compared to other migratory insects (Hobson et al. 2012). The exceptional ability for long-distance migration (possibly related to passive dispersal mediated by human transport or wind) explains why *P. flavescens* is even present on one of the world’s most remoted islands, the Easter Island, Chile (Samways and Osborn 1998). The analysis of whole mitogenomes from migratory as well as resident *P. flavescens* populations would be a promising approach to further study the genetic pathways linked to the migratory behavior in this species and would substantially extend insights gained from previous single marker gene analyses (Troast et al. 2016; Alvial et al. 2019). We here report on the first mitogenome of *P. flavescens* from a migratory population in Tanzania.

A standard phenol–chloroform protocol by Hadrys et al. (1992) was used to extract total genomic DNA from a mid-leg of an individual collected at Rufiji River, Tanzania (−7.854316; 38.428008) in the frame of the BIOTA AFRICA project in 2002. The tissue and DNA samples (PFLRRT32002) are stored in the Institute of Animal Ecology, University of Veterinary Medicine Hannover, Foundation, Germany (contact person is the first author). PCR primers were designed based on transcriptomic data (PRJNA239794) from a migratory *P. flavescens* specimen from China. Sanger sequencing was conducted at DNA Analysis Facility at Yale University, New Haven, CT and Geneious version 8.1.8 (https://www.geneious.com) was used to assemble the overlapping mitogenome sequences. For mitochondrial genome annotation the MITOS WebServer (Bernt et al. 2013) was used and results were manually checked using BLAST (Altschul et al. 1990). The phylogenetic position of *P. flavescens* was assessed in the context of all available anisopteran mitogenomes to date (10/2020) mined from GenBank. Protein-coding genes (PCGs) were aligned using MAFFT version 7.017 (Kumar et al. 2016) and subsequently concatenated. Maximum likelihood (ML) analyses were performed using the best-fitting model GTR + F + I + G4 under the Bayesian information criterion detected via ModelFinder (Kalyaanamoorthy et al. 2017) with IQ-tree keeping default settings (Nguyen et al. 2015). *Ischnura elegans* (Zygoptera, NC_031824) served as outgroup.

The complete circular mitochondrial genome sequence of *P. flavescens* (GenBank Accession No. MW256717) with a length of 14,853 bp is the most compact known mitogenome among dragonflies (Anisoptera). The standard metazoan gene content of 37 genes is identically arranged as in other odonate mitochondrial genomes (e.g. Simon and Hadrys 2013; Feindt et al. 2016). The overall base frequency is AT-biased (72.7%). Regarding invertebrate mitochondrial start codons, ATT (*nad2*, *atp8),* ATA (*cox1*, *nad3*, *nad4*, and *atp6*), ATC (*nad6*), ATG (*cob*, *cox2*, *cox3*, and *nad4l*), and TTG (*nad1*) standard codons are found. All PCGs possess TAA as complete stop codon except for *cox2* and *nad5*, which possess a single T which is likely completed by post-transcriptional polyadenylation. All tRNA secondary structures exhibited a typical clover-leaf structure. However, *trnS1* lacked the dihydrouridine (DHU) arm. The AT rich control region (CR) is 171 bp long and therefore the shortest observed CR among all Anisoptera.

The phylogeny of all available anisopteran mitogenomes is shown in Figure 1. All families within Anisoptera were consistently supported as monophyletic groups if represented by more than one species. Within the Libellulidae, *P. flavescens* is placed as a sister to *Nannophya pygmaea.*

**Figure 1.**
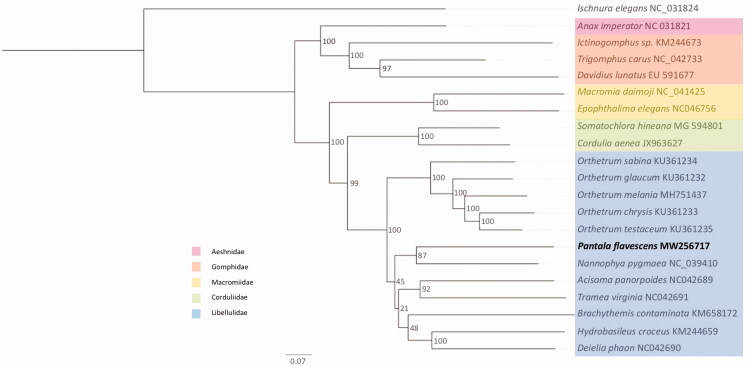
Maximum likelihood (ML) tree for 20 Anisoptera using 13 concatenated mitochondrial protein-coding genes (11,375 bp). Maximum likelihood bootstrap support values are given at each node. The Zygoptera *Ischnura elegans* served as outgroup and the here presented mitogenome of *Pantala flavescens* is given in bold.

The first complete mitochondrial genome of *P. flavescens* from a migratory population from Tanzania paves the way for further comparative approaches, which should include both, other migratory populations (e.g. from Asia or South America) as well as the so far only known non-migratory population from Easter Island, Chile. Such comparative mitogenomic approaches might help to further reconstruct dispersal routes and migration history of this unique odonate model species.

## Data Availability

The data that support the findings of this study are openly available in GenBank of NCBI at https://www.ncbi.nlm.nih.gov, reference number MW256717.
